# Supplementary material for: Learning curve and surgical outcome of robotic assisted colorectal surgery with ERAS program
Source: Sci Rep. 2022 Nov 29;12:20566. doi: 10.1038/s41598-022-24665-w (PMC9709162; doi:10.1038/s41598-022-24665-w)
Supplement: Supplementary file 1 — Supplementary Information 1. [file 41598_2022_24665_MOESM1_ESM.zip › LSC Raw data-Ñ[▒K/142/N_ñw╝╨░O▒Kñσ.pdf]

W83 / W83

臺中榮民總醫院
手術室護理記錄

[redacted]

基本資料

- 室別： 二醫手術室-40室，手術科別： CRS，手術類別： 常規，日期： 2021/10/20
- 進入時間： 1415 ； 麻醉開始時間： 1415 ； Time Out時間： 1516 ； 劃刀時間： 1518 ； 手術結束時間： 1815 送出時間： 1825 ；
- 麻醉方式： GE
  診斷：S colon ca 30 cm FAV
  手術名稱：Laparoscopic Anterior resection and anastomosis - malignant
  手術局部用藥：nil
  術前預防性抗生素：
  - 抗生素名稱一： Veterin 1GM IVD/AR
  - 時間： 1500
  - 上次簽章: 許育庭

  ，追加預防性抗生素：
- 手術醫師： 林俊余，陳璿夫，林順鴻，陳凱祥
- 麻醉醫師： VS 賴慧卿
-
  - 刷手護士A ： 羅渝軒，時間： 1415，接替刷手一： 顏育?，時間： 1730，接替刷手二： 羅渝軒，時間： 1800
  - 流動護士A ： 許育庭，時間： 1415，接替流動一： 梁凱雯，時間： 1700，接替流動二： 羅渝軒，時間： 1800

皮膚狀況

A-V Shunt： 無
術前： 完整
記錄者： 上次簽章: 許育庭
術後： 完整
記錄者： 羅渝軒

手術準備

手術臥式 單一手術臥式

-
  - 手術臥式： 截石臥式
  - 使用附件： 溫毯 38 ℃，矽膠枕頭，頭圈，腳架，其它： 頭架
  - 皮膚準備： 10%Aq Betadine，Hibiscrub，2% Chlorhexidine
  - 電燒： 使用電燒: 電極片位置： 其它： 背部迴路電極墊，記錄者： 上次簽章: 許育庭

止血帶T： 無

植入物

- 植入物名稱/號碼/數量/部位：1.ETHICON LIGACLIP(LOT:U94864)/12mm/20釘/colon
  2.ETHICON ENDO-GIA60(LOT:V94D3F)/60mm/壹釘/colon

，記錄者： 上次簽章: 許育庭

標本送檢

- 細菌培養 無
- 細胞學 無
- 冰凍切片 無
- 病理標本
  組織名稱一：PA LN
  ，
  組織名稱二：S-COLON
  ，
  組織名稱三：Distal cut end

，更多組織名稱
  - 組織名稱四： IMA Root
- 截肢 無

標本交付 標本： 肆 袋 ，標本： 零 盒 ，標本： 零 瓶 ，細胞學： 零 管 ，Culture Tube： 零 管 ，被交付醫師： R 林順鴻 ，

- 記錄者： 羅渝軒
- 時間： 1800

傷口術後敷料

傷口敷料： 傷口敷料： 無

- 請於圖上標示部位：

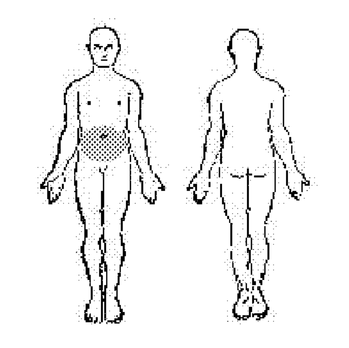

腹部参切口

- 傷口敷料： Mepore

導管

導管	天數	導管資訊	插入日期	插入時間	更換日期	拔除日期	拔除時間	拔除原因	預計拔除/更換	預計拔除/更換日期
Hemo Vac(1)	1	號碼:19 Fr.; 部位:骨盆腔; 註記:右下腹;	2021/10/20	1800						
Foley(1)	1	號碼:16 Fr.; 部位:膀胱; 註記:水球10cc;	2021/10/20	1505						
Sump NG Tube(1)	1	號碼:16 Fr.; 深度:55cm; 部位:胃;	2021/10/20	1455						
A-line (1)	1	號碼:20 G; 部位:橈動脈; 註記:右手;	2021/10/20	1440						

用物計數

- 手術中出血量： 微量 c.c.
- 手術中尿量： 100 c.c.
-
  - 其它： 無

用物計數： 多切口同時縫合

- 1.器械： 正確 ，2.縫針： 正確 ，3.刀片： 正確 ，4.紗布： 正確 ，5.腹部墊： 未使用 ，6.紗球： 未使用 ，7.PEANUTS ： 未使用 ，8.紗條： 未使用 ，9.絲帶： 未使用 ，10.針頭： 未使用 ，11.Vessel loop： 未使用 ，12.Boots： 未使用 ，13.Jelco： 未使用 ，14.棉條： 未使用 ，
  - 第一次：
    - 刷手護士 ： 羅渝軒
    - 流動護士： 上次簽章: 許育庭
    - 時間： 1650

，
  - 第二次：
    - 刷手護士 ： 羅渝軒
    - 流動護士： 上次簽章: 梁凱雯
    - 時間： 1715

，
  - 第三次：

- 刷手護士 ： 顏育?
- 流動護士： 上次簽章: 梁凱雯
- 時間： 1745

備註

備註： NIL

- 記錄者： 上次簽章: 許育庭
- 時間： 1700

填寫護理人員 羅渝軒

正式摘要

W83 / W83

doctorRank:B
